# Comparison of Petrifilm^TM^ AC and pour plate techniques used for the heterotrophic aerobic bacterial count in water

**DOI:** 10.1093/femsle/fnae029

**Published:** 2024-04-29

**Authors:** Faith Mkhwanazi, Tshilidzi Mazibuko, Olivia Mosoma, Malefaso Rathebe, Mrudula Patel

**Affiliations:** Infection Control, Charlotte Maxeke Johannesburg Academic Hospital National Health Laboratory Service, 7 York Road, Parktown, Johannesburg 2193, Gauteng, South Africa; Infection Control, Charlotte Maxeke Johannesburg Academic Hospital National Health Laboratory Service, 7 York Road, Parktown, Johannesburg 2193, Gauteng, South Africa; Infection Control, Charlotte Maxeke Johannesburg Academic Hospital National Health Laboratory Service, 7 York Road, Parktown, Johannesburg 2193, Gauteng, South Africa; Infection Control, Charlotte Maxeke Johannesburg Academic Hospital National Health Laboratory Service, 7 York Road, Parktown, Johannesburg 2193, Gauteng, South Africa; Infection Control, Charlotte Maxeke Johannesburg Academic Hospital National Health Laboratory Service, 7 York Road, Parktown, Johannesburg 2193, Gauteng, South Africa; Department of Clinical Microbiology and Infectious Diseases, School of Pathology, Faculty of Health Sciences, University of the Witwatersrand, Private Bag 3, Wits, Johannesburg 2050, South Africa

**Keywords:** Petrifilm, water, TPC, aerobic bacteria, bacterial counts, heterotrophic

## Abstract

Heterotrophic bacteria are commonly found in water samples. While these Heterotrophic Bacterial/Plate Counts (HPC) do not necessarily indicate a health hazard, high counts provide a good indication of the efficiency of water disinfection and integrity of distribution systems. The aim of this study was to compare the Petrifim^TM^ AC method to the pour plate technique for the testing of HPC in water samples. Artificially contaminated (192 samples) and natural water samples (25) were processed using two methods. Both methods accurately detected high, medium and low counts of HPC, producing average Z scores between −2 and +2. Paired-wise student *t*-test and correlation coefficient showed nonsignificant differences between the results of two methods. Acceptable repeatability and reproducibility was obtained using both the methods. Uncertainty of measurement for Petrifilm^TM^ AC and pour plate method was found to be 2.9% and 5.4%, respectively. Petrifilm^TM^ AC proved to be robust at 33°C and 37°C. In conclusion, Petrifim^TM^ AC, which is easy to process, read, and less time consuming, proved to be comparable to the conventional pour plate method in establishing HPC in water. In addition, Petrifim^TM^ AC requires less space for the processing and incubation, generate small volume of waste for disposal, and requires no equipment, except for the incubator.

## Teaser

Petrifilm^TM^ is a simple, easy, and effective method for establishing heterotrophic bacterial counts in water.

## Introduction

According to the World Health Organization (WHO) drinking-water should be suitable for human consumption, washing/showering, and the preparation of food. Exposure to water and its constituents can occur through ingestion, contact, and inhalation. Therefore, drinking water should be microbiologically safe (WHO 2023). In 2022, according to WHO, 6 billion people used safely managed uncontaminated drinking water and 2.2 billion people used untreated water. While the untreated water is known to transmit pathogens; with a mismanagement of treated water and supply chain broken, treated water can also become a source of infection (WHO 2023, UN-Water 2021). For example, a systematic review containing studies from high-income countries mainly from north America, showed that the vast majority of residents (≥90%) reportedly have high access to safely managed drinking water, and yet the burden of water-related gastrointestinal illness risks was ~2720 annual cases per 100 000 population (Lee et al. 2023).

Microorganisms can grow in water and on surfaces in contact with water, such as distribution pipes, domestic plumbing, and vending machines. Commonly found microorganisms are heterotrophs, which require organic carbon for growth. These include bacteria, yeasts, and mould. A wide range of microorganisms, which require varied nutrients, physiological conditions, and incubation time. Therefore, at a given time and growth condition used, microbial recovery will include a fraction of natural microbiota of water, as well as contaminants (Allen et al. 2004).

With regards to the total heterotrophic plate counts (HPC) in treated potable water, countries have different directives (Saxena et al. 2015), and the acceptable counts varies from 10 to 1000 cfu/ml of water. In general, HPC have no health burden and the test cannot stand alone. Additional tests, such as total coliform or the counts of faecal coliforms and *Escherichia coli* counts, are required to determine the health hazard. Nevertheless, counts of heterotrophic bacteria provides a good indication of efficiency of water disinfection and the integrity of distribution systems. Constant surveillance can show early detection of possible presence of external contamination of water (WHO, EPA 2024, Canadian drinking water, EN directive 2020). A very high number of heterotrophic bacteria, however, does indicate possible water contamination and the possible presence of health hazards. In South Africa, drinking water guidelines and national standards allows Heterotrophic Bacterial/Plate Count (HPC) of ≤1000 cfu/ml in drinking water (SANS 241-1 2015). Counts higher than that are described to have increased risk of infectious disease transmission, particularly respiratory infections including Legionella, and opportunistic infections in immunocompromised individuals, and nosocomial infections including wound infections, urinary tract infections, and postoperative infections. In addition, high counts of HPC can lead to biofilm formation in pipes leading to further establishment of microorganisms, which can also cause foul taste and aesthetically reduced quality of water (Allen et al. 2004, WHO 2022).

Many methods are described for the enumeration of HPC, such as membrane filtration, pour plate, spread plate, and fluorescent substrate technique, each with advantages and disadvantages (Allen et al. 2004). The pour plate method is most commonly used (Shakoor et al. 2018, Amin et al. 2019). Ready-to-use dehydrated media have been developed and commercialized as Petrifilm^TM^ AC. It is widely used for total bacterial counts in food and milk samples. Petrifilm^TM^ AC, which is used for aerobic bacterial counts, has not been tested and validated for the water samples. The aim of this study was to compare the conventional pour plate technique to Petrifilm^TM^ AC and validate the Petrifilm^TM^ AC to test water samples for the total bacterial counts.

## Methods

### Water samples and spiking with bacteria

Chlorinated tap water was collected from a drinking water tap into 1 l glass bottles. Borehole water (1 l) was collected from the Parktown, Johannesburg region. Bottled water was obtained from a supermarket. Tap water and borehole water samples were autoclaved. Sodium thiosulfate (1%), which is generally used to neutralize the residual chlorine in the samples, was added to one set of tap water. This was to establish if it had any effect on the final HPC results.

Cultures of *E. coli* ATCC 25922, *Bacillus subtilis* ATCC 6633, *Pseudomonas aeruginosa* ATCC 27853, *Enterococcus faecalis* ATCC 21922, and *Candida albicans* ATCC 90028 were obtained from the Infection Control laboratory, National Health Laboratory Services (NHLS), South Africa. For the use of these stock cultures, an ethical waiver was obtained from the Human Research ethic Committee, University of The Witwatersrand (W-CBP-230711-01). Raw material for the culture media and reagents were purchased from Merck, South Africa. Cultures were grown on blood agar plates at 37°C for 24–48 h, and purity was established. One isolated colony from each of the culture plates was emulsified into 10 ml sterile distilled water to obtain McFarland standard of 0.5 containing ~10^8^ cfu/ml. Serial 10-fold dilutions (up to 10^−6^) were prepared using 9 ml buffered peptone water. Dilutions 10^−3^, 10^−4^, and 10^−5^ were selected for further testing. Original suspension of 0.5 was also serially diluted and each dilutions were plated onto TSA agar plates to obtain an accurate count total bacteria, which were considered as expected counts.

Water samples were inoculated with selected dilutions to obtain ~1000, 100, and 10 cfu/ml of water samples representing high, medium, and low quantities of test counts. Uninoculated samples were also processed as negative controls. Sterility of all the media, including Petrifilm^TM^ AC, was established.

### 3M^TM^ Petrifilms aerobic plate count method (AC)

Tests were performed as per manufacturer’s instructions (3M, South Africa). Two plates per sample were used. Briefly, 1 ml of sample was pipetted perpendicularly into the centre of the bottom film containing dehydrated media and spread using a spreader as provided with the Petrifilms^TM^ AC. Plates were kept on the bench for 15 min and allowed to solidify and set. Plates were incubated at 35 ± 2°C for 48 h in a closed container with a moisture pack to prevent dehydration. Humidity of these containers was not measured in accordance with manufacturer’s instructions. Red colonies were counted regardless of size and intensity. Preferred counting range was 10–300 cfu/ml, however, colonies more than the upper range were counted as estimated counts. Number of colonies in one square were counted and multiplied by 20 to obtain the total number per plate, hence 1 ml of sample. The average of the counts of the two plates was taken as a final count.

### Pour plate method

Pour plate method is one of the oldest techniques used for the total bacterial counts. Briefly, 1 ml of sample was placed in the centre of a sterile Petri dish, molten cooled (45°C) plate count agar (Merck, South Africa) was poured over it and mixed gently. The media was allowed to solidify and set. Plates were inverted and incubated at 37°C for 48 h (Jackson et al. 2000). Clear, white, and opaque colonies on the surface of the agar as well as into the agar at various levels, were counted. Two plates per sample were used. The average of the counts of the two plates was taken as a final count.

### Validation procedure

The validation procedure described in TR 28 document was used with minor modifications (TR 28-01 2014). This document defines the technical requirements for quality control, quality assurance, and the validation of methods in microbiological testing laboratories. Accuracy was established by calculating Z scores using the complete data (16 results + inoculum). An acceptable range was set at −2 to +2. Repeatability was calculated using seven results obtained by one analyst on the same day. Reproducibility was calculated using nine results obtained by three analyst on three different days. Results were compared between analysts. Relative standard deviations (RSD) were calculated for the repeatability and reproducibility. Acceptable RSD was set at ≤0.1. Uncertainty of measurement was calculated using reproducibility results. Robustness was established using two different incubation temperatures (33°C and 37°C).

### Natural samples

Natural samples of potable water (25) received by the Public Health Laboratory, NHLS, Johannesburg for routine testing were processed in parallel using both the methods. Results were analysed using correlation coefficient.

### Participation in an external quality assurance

To further establish the reliability of the Petrifilm^TM^ AC, our laboratory participated in an external quality assurance, where samples were provided by Axio QWAS distribution, LGC Profeciency testing, 1 Chamberhall Business Park, Chamberhall Green Bury, UK. In total, 21 distributions were processed using the Petrifilm^TM^ AC method between November 2017 and June 2023. The average number of laboratories participated per distribution is 204 and the results were analysed by the distributor. Results were received in the form of Z scores, with an acceptable range between −2 and +2.

## Results

### Accuracy and the comparison of both methods

Results of spiking inoculum (expected results) were compared to the results obtained by all four analysts. This was performed for all the matrices, two methods, and three concentrations. All the counts were converted into log_10_ and analysed. Mean ± SD counts for Petrifilm^TM^ AC for the high, medium, and low concentrations were 3.08 ± 0.13, 2.44 ± 0.12, and 1.68 ± 0.13, respectively. Whereas, for the pour plate method, they were 3.14 ± 0.13, 2.45 ± 0.12, and 1.58 ± 0.16, respectively. The Z scores of each test results were found to be between −2 and +2 (Table 1). This suggested that both the methods produced accurate results for all the matrices at high, medium, and low concentration of bacteria. When the results obtained with Petrifilm^TM^ AC were analysed with the results of pour plate technique, it was shown that the results are comparable with mean Z-score between −2 and +2 (Table 2). The correlation coefficient (0.95) was acceptable (Figure S1 and Table S1) and the paired *t*-test showed that there was no difference in the counts obtained from the two methods (*P* > .05).

**Table 1.** Accuracy of the two methods for the aerobic plate count in water.

		Mean Z score (*n* = 16)	
Matrix	Concentration	Petrifilm^TM^	Pour plate
Tap water with thiosulfate White cap bottles	High	0.02	−0.01
	Medium	−0.02	0.01
	Low	−0.04	0
Tap water without thiosulfate Red cap bottles	High	0.02	−0.09
	Medium	−0.02	0
	Low	−0.08	0
Borehole water	High	0	0
	Medium	0	0
	Low	0.04	0
Bottled water	High	0	0
	Medium	0	0
	Low	0	0

**Table 2.** Comparison and accuracy using combined results of both the tests for aerobic plate count test in water and linearity.

Concentration	Petrifilm^TM^ and pour plate methods mean ± SD (*n* = 256)
High	Z score 0 ± 1
Medium	Z score 0 ± 1
Low	Z score 0 ± 1
Correlation: r	0.97
Paired *t*-test (*P-*value)	0.12*

* nonsignificant difference in methods.

### Repeatability and reproducibility

Repeatability and reproducibility are important as it indicates that the methods are robust. If the confounding factors and competencies of operators are considered, it produces accurate results. For the repeatability, results obtained by one analyst (seven readings) for all four matrices, both the methods, and all three concentrations (high, medium, and low) were analysed for RSD. The results were found to be in an accepted range of ≤0.1 RSD as per Table 3. For the reproducibility, results obtained by three analyst on three different days were compared and analysed for relative standard deviation (RSD_RC_). Acceptable mean RSD_RC_ of ≤0.1 were obtained for both the methods (Table 4).

**Table 3.** Repeatability of Petrifilm^TM^ and pour plate methods for the aerobic plate counts in water.

		RSD	
Matrix	Concentration	Petrifilm^TM^ (*n* = 7)	Pour plate (*n* = 7)
Tap water with thiosulfate	High	0.01	0.01
White cap	Medium	0.01	0.02
	Low	0.02	0.02
Tap water without thiosulfate	High	0.01	0.03
Red cap	Medium	0.02	0.02
	Low	0.01	0.02
Borehole water	High	0.01	0.02
	Medium	0.04	0.05
	Low	0.06	0.1
Bottled water	High	0.01	0.03
	Medium	0.02	0.04
	Low	0.03	0.05

**Table 4.** Reproducibility of Petrifilm^TM^ and pour plate methods for the aerobic plate count in water.

Reproducibility	Petrifilm^TM^ (*n* = 36)	Pour plate (*n* = 36)
RSD_RC_	0.029	0.054
Minimum RSD_R_	0.003	0.003
Maximum RSD_R_	0.06	0.155

### Uncertainty of measurement and robustness

Uncertainty of measurement was calculated using results of reproducibility (RSD_RC_) for both the methods including all the matrices. These results may vary from laboratory to laboratory. In our laboratory, for Petrifilm^TM^ AC, with counts of 100 cfu/ml, the uncertainty was found to be between 87 and 114 cfu/ml of water (2.9%). For pour plate, with counts of 100 cfu/ml, the uncertainty was found to be between 78 and 128 cfu/ml (5.4%). Robustness was determined only for the Petrifilm^TM^ AC method because the aim of this study was to validate this method. Petrifilm^TM^ AC tests were performed in two duplicate sets. One set was incubated at 33°C and the other at 37°C. The rationale was determine the effect on final results should the incubator temperatures fluctuate by 2°C either way. The results showed that there was no difference in the results obtained at 33°C and 37°C in Petrifilm^TM^ AC (Table 5).

**Table 5.** Robustness of Petrifilm^TM^ method for the aerobic plate count in water.

		RSD—Petrifilm^TM^
Matrix	Concentration	33°C vs. 37^o^C
Tap water with thiosulfate White cap	High	0.01
	Medium	0.01
	Low	0.09
Tap water without thiosulfate Red cap	High	0.01
	Medium	0.01
	Low	0.08
Borehole water	High	0.00
	Medium	0.01
	Low	0.07
Bottled water	High	0.00
	Medium	0.01
	Low	0.07

### Effect of sodium thiosulfate

When the results of water samples, with and without sodium thiosulfate, were compared, no significant difference in the HPC was found; with *P*-values of > .05 (Table 6)

**Table 6.** Comparison of HPC in the water samples with and without sodium thiosulfate.

		Mean ± SD cfu/ml (*n* = 16)		
Method	Concentration	Without Na_2_S_2_O_3_	With Na_2_S_2_O_3_	*P*–value
Petrifilm^TM^	High	1245 ± 417	1223 ± 386	0.88
	Medium	306 ± 84	300 ± 106	0.87
	Low	60 ± 8	60 ± 11	0.84
Pour plate	High	1302 ± 319	1294 ± 319	0.95
	Medium	344 ± 78	307 ± 78	0.19
	Low	41 ± 11	48 ± 11	0.06

### Natural samples

Out of 25 samples, 15 samples colony counts were impossible to count using both the methods (too numerous to count). A total of 10 samples provided measurable counts. The mean count and the range for Petrifilm^TM^ AC was 167 cfu/ml and 1–710 cfu/ml, respectively. Whereas, for the pour plate method, the mean and range were 176 cfu/ml and 5–736 cfu/ml, respectively. For these 10 results, the correlation coefficient was 0.99 (Table S2).

### Participation in an external quality assurance

All 21 rounds of distribution received acceptable Z scores between −1 and +1 using Petrifilm^TM^ AC (Figure S2), further establishing accuracy of the Petrifilm^TM^ AC method.

## Discussion

Aerobic plate count, which is a synonym for heterotrophic plate count or total bacterial counts, has a long history of use in water microbiology. HPC measurements indicates the effectiveness of water treatment processes, post-treatment contamination, and regrowth of organisms. The pour plate technique is one of the oldest methods developed for the determination of HPC in water. Media containing basic nutrient, such as tryptone and yeast extract is used, which supports the growth of wide variety of bacteria that require organic carbon for growth. It is an effective, validated, well-recognized, and widely used method. However, it can be time consuming especially in very busy laboratories that process high volumes of water samples. 3M^TM^ has developed dehydrated agar plates containing a water-soluble gelling agent, nutrients, and indicator that facilitates enumeration of HPC. A built-in grid facilitates counting of colonies, to provide fast, precise, and consistent results. It has been extensively used for testing food samples, however, it has not been studied for the testing of water samples. Results in this study showed that Petrifilm^TM^ AC method is comparable to the pour plate method, with the Z score around zero when the results of both the methods were analysed together for the high, medium, and low counts. The correlation coefficient was also acceptable at 0.95. In addition, international proficiency testing results were also comparable to ~200 laboratories that used many different methods. In contrast, when Schraft and Watterworth (2005) compared the Petrifilm^TM^ AC method to the membrane filtration to test water, they found a significant difference in the counts, with the counts on Petrifilm^TM^ AC being 0.5 log lower than membrane filtration technique. In their study, only natural samples were processed, whereas in our study, sterile samples were inoculated with a mixture of organisms to cover a range of counts, as well as natural samples.

Although Petrifilm^TM^ AC is validated for the food and milk testing and well-studied (McAllister et al. 1988, Curiale et al. 1990), it has not been studied for the water samples. The reason could be, although HPC has significance in the water samples, there are no legislated acceptable counts available in many countries because it is not a standalone test (Bartram et al. 2003, Allen et al. 2004, Saxena et al. 2015). For potable waters, coliform and *E. coli* counts are required as additional tests. Several epidemiological studies have failed to establish the correlation between the HPC in drinking water and gastrointestinal infections (Calderon and Mood 1988, Payment et al. 1991, Hellard et al. 2001). This suggests that HPC is not a health-related indicator, but it is a process indicator (disinfection and reticulation). For the quality indicator of water service delivery, this test can easily be used. In South Africa, HPC requirement in the potable water is legislated with the acceptable counts of ≤1000 cfu/ml of drinking water, therefore this test is extensively used locally (SANS 241-1 2015). Counts between 100 and 1000 cfu/ml are indicative of some contamination or inadequate treatment, but counts >1000 cfu/ml has increased risk of infectious disease transmission (Department of Water Affairs and Forestry 1996), particularly respiratory infections and nosocomial infections. United States Environmental Protection Agency allows ≤500 cfu/ml of HPC in ground and drinking water (EPA).

Petrifilm^TM^ AC is a simple method that requires very short processing time when compared to the pour plate technique (1–2 min vs. 30 min). Dehydrated media is ready to use and it requires less handling, thus reducing chances of contamination compared to the pour plate method, where melting, cooling, and pouring of media is required. Petrifilm^TM^ AC do not require any equipment. Since these plates are compact, laboratory space for processing and the incubator space required is also less than pour plate method. Thickness of Petrifilm^TM^ AC is 0.1 cm, and therefore can be stacked in large quantities for incubation, whereas Petri dishes used for the pour plate have a thickness of 1.5 cm that require more space during processing of samples and incubation. Waste disposal can also be expensive because it is calculated per weight of the waste. Petrifilm^TM^ AC weighs 2.8 g with a sample whereas pour plate weighs 32.7 g. One of the additional disadvantages of the pour plate method is that the agar has to be used at precisely 45°C, any higher than that the temperature may kill some of the bacteria. Agar at lower temperatures than 45°C may start solidifying at room temperature, creating lumps and compromising even distribution of colonies into the agar, thereby compromising colony counts. In Petrifilm^TM^ AC, although the colony counts are performed in 5-cm diameter of rehydrated media film compared to the 9-cm diameter surface of pour plate agar, counting colonies is easier due to the pink colour of the colonies. In the pour plate method, mostly white colonies are on the surface and submerged into the pale colour media, making it difficult to read the results. Although Petrifilm^TM^ AC has a counting grid, in the case of higher counts, counting colonies can be a problem. Therefore, manufacturer’s recommended accurate count is 300 cfu/ml/plate. Above these counts, colony counts are possible but may not be accurate, and therefore reported as an estimated counts.

Both the methods allow accurate counts up to 300 cfu/ml of sample, thereafter if possible, higher counts can be obtained by diluting the sample. Presence of bacteria that cause liquefaction of agar and those that have the ability to spread on the agar surface can create interference in counting colonies especially in the Petrifim^TM^ AC (Blackburn et al. 1996).

Results also showed that the repeatability and reproducibility of Petrifilm^TM^ AC was acceptable, meaning the results are not affected by the conditions and analysts. Robustness was studied using variation in only one condition (temperature) due to the resource constraints. Nevertheless, Petrifilm^TM^ AC proved to be acceptable in the presence of two incubation temperatures. However, Ellender et al. (1993) found higher HPC in food samples when Petrifilm^TM^ AC were incubated at room temperature compared to the 35°C incubation. Perhaps, the nutrients present in the food samples influenced the bacterial counts. Water samples generally are low in any form of nutrient, therefore the results were not affected.

In conclusion, Petrifilm^TM^ AC proved to be comparable to the widely used traditional pour plate method. With many advantages, Petrifilm^TM^ AC proved to be an accurate, repeatable, reproducible, and robust method for establishing heterotrophic bacterial counts in water.

## Supplementary Material

fnae029_Supplemental_File
